# Cognitive enrichment in a social setting: assessing the use of a novel food maze in sanctuary-housed chimpanzees

**DOI:** 10.1007/s10329-022-00996-0

**Published:** 2022-07-18

**Authors:** Maria Padrell, Federica Amici, Maria Pau Córdoba, Miquel Llorente

**Affiliations:** 1 Departament de Psicologia, Facultat d’Educació i Psicologia, Universitat de Girona, Plaça Sant Domènech 9, 17004 Girona, Spain; 2 Research Department, Fundació Mona, 17457 Girona, Spain; 3 Department of Comparative Cultural Psychology, Max Planck Institute for Evolutionary Anthropology, 04103 Leipzig, Germany; 4 Faculty of Life Sciences, Institute of Biology, University of Leipzig, 04103 Leipzig, Germany; 5 Institut de Recerca i Estudis en Primatologia, IPRIM, 17246 Santa Cristina d’Aro, Spain

**Keywords:** Behavior, Chimpanzees, Cognitive enrichment, Tool use, Welfare

## Abstract

Foraging devices are effective enrichment tools for non-human primates, as they provide both cognitive and manipulative stimulation that may enhance these animals’ welfare. We assessed the behavioral effects of a novel tool-based enrichment on 14 chimpanzees (*Pan troglodytes*) housed at Fundació Mona (Girona, Spain). The device consisted of a vertical maze filled with food rewards, which chimpanzees could extract by using tools. We conducted behavioral observations in two conditions over an approximately 2.5-month period: when the food maze was loaded (12 enrichment days), and when it was empty (12 baseline days). Data were collected using 2-min scan sampling and untimed-event focal sampling during two daily sessions of 80 min each. We expected that the chimpanzees’ interest in the enrichment would decrease over time, but that its use would be linked to an increase in the occurrence of species-typical behaviors, a reduction in negative indicators of welfare, and changes in social behaviors. We found that participation widely varied among subjects, being higher in females and decreasing through time. Furthermore, participation was linked to an increase in tool use and a decrease in inactivity, but also to an increase in aggression-related behaviors. In contrast, participation had no effect on the occurrence of abnormal behaviors, social proximity or affiliation-related behaviors. Finally, we detected an increase in self-directed behaviors only when subjects actively interacted with the device. We conclude that, in future studies, these types of devices should be evaluated for longer periods of time and more attention should be paid to individuals’ preferences and abilities.

**Supplementary Information:**

The online version contains supplementary material available at 10.1007/s10329-022-00996-0.

## Introduction

Behavioral diversity and species-typical behaviors, also referred to as “ethological needs,” are key concepts related to animal welfare (Browning 2019; Hughes and Duncan 1988; Miller et al. 2020). However, captive settings often lack sufficient complexity to allow the expression of a species-typical behavioral repertoire (Mallapur 2008; Newberry 1995; Young 2003). For this reason, environmental enrichment has become a key component of the management of captive animals (Maple and Perdue 2013), as it is considered an important means of improving animal welfare by providing opportunities for physical, affective and cognitive stimulation (Fernández and Martin 2021; Hoy et al. 2010; Mellor 2015). The extensive variety of enrichment strategies used in non-human primates includes sensory stimulation (Carter et al. 2021; Vaglio et al. 2021), social housing (Chipangura et al. 2020), motor or manipulative engagement (Costa et al. 2018), and more recently, cognitive stimulation (Coleman and Novak 2017; Dutton et al. 2018; Lutz and Novak 2005), which also includes the use of digital electronic devices (Clark 2017; Clark et al. 2019; Gray et al. 2018; Grunauer and Walguarnery 2018; Kim-McCormack et al. 2016). In fact, cognitive enrichment has become increasingly popular in zoos, sanctuaries and even farms, where problem-solving opportunities can enhance animal welfare (Clark 2017; Meehan and Mench 2007). Cognitive enrichment can be defined as an enrichment which “(1) engages evolved cognitive skills by providing opportunities to solve problems and control some aspect of the environment, and (2) is correlated to one or more validated measures of well-being” (Clark 2011, p. 6).

Cognitively stimulating environments may be particularly important for captive non-human primates, and more specifically for great apes due to their behavioral, affective and cognitive complexity (Clark 2011). In the wild, great apes continuously face complex ecological and social problems that require complex perception, understanding and decision-making skills (Morimura 2006), so that psychological challenges are likely inherent in their nature. This could explain why chimpanzees, for example, reportedly engage in problem-solving activities even when no reward is involved (Clark and Smith 2013). Furthermore, great apes often explore novel objects (Paquette and Prescott 1988), possess highly developed manipulative skills (Paquette and Prescott 1988; Torigoe 1985), and use and create tools in captive environments (Motes-Rodrigo and Tennie 2021). Therefore, the introduction of novel devices or tasks promoting such behaviors may be a particularly successful enrichment strategy for these species.

Furthermore, non-human primates spend considerable amounts of time foraging for and eating food in the wild, e.g., in chimpanzees, up to 18.8–60% of their time (Boesch and Boesch-Achermann 2000; Doran 1997; Inoue and Shimada 2020; Pruetz and McGrew 2001; Yamanashi and Hayashi 2011). However, captive animals are usually provided with food directly, in ways that require minimal effort. Thus, captive chimpanzees typically spend less time foraging than their wild counterparts (Inoue and Shimada 2020; Yamanashi and Hayashi 2011), and this may be linked to reduced behavioral expression and competence, and heightened negative emotional states (Špinka and Wemelsfelder 2011). For these reasons, enrichment activities were employed in several studies with the focus on increasing opportunities for foraging (Baker 1997; Bloomsmith et al. 1988; Maki et al. 1989; Reinhardt 1993), and included food hidden inside boxes, pipes, tubes or balls (Brooks et al. 2021; Gronqvist et al. 2013; Nash et al. 2021) that could only be extracted by manipulating the objects in a specific manner (e.g., poking, shaking, rotating). The sophistication of a device can be altered to vary the complexity of the problem-solving skills required, but it should provide both manipulative and cognitive stimulation to non-human primates (Dutton et al. 2018), who usually show an interest in food-associated enrichments and motivation to solve puzzles for food rewards (Cheyne 2009; de Rosa et al. 2003; Shohat et al. 2019), even when highly valued foods are not used (Brooks et al. 2021). Furthermore, as foraging devices make food more difficult to obtain, primates spend more time on these activities and less time inactive or engaged in abnormal behaviors (Brent and Eichberg 1991). Similarly, these types of enrichment can increase the general activity of the group over longer periods, even if only a few individuals actively manipulate the devices (Csatádi et al. 2008; Jones and Pillay 2004). Most food-based enrichments for great apes involve toys or objects like boxes, pipes, tubes or balls (Bloomsmith et al. 1990, 1991; Brent and Stone 1998; Lambeth and Bloomsmith 1994; Pruetz and Bloomsmith 1992), i.e., relatively unsophisticated devices, partly because of time constraints (e.g., time to design or manufacture the devices) and limited finances (Hall et al. 2021).

Several studies have employed foraging devices that require tool use in captive great apes, particularly chimpanzees (Celli et al. 2004, 2003; Clark and Smith 2013; Llorente and Campi 2014; Maki et al. 1989; Morimura 2003; Nash 1982; Padrell et al. 2021; Yamanashi et al. 2016; Zaragoza et al. 2011). These devices often simulate behaviors such as termite-fishing, ant-dipping or ant-fishing, which are commonly observed in the wild (Boesch and Boesch 1990; Goodall 1986; Jones and Sabater Pi 1969). In general, these activities enhance chimpanzee welfare by increasing species-typical behaviors and decreasing abnormal behaviors and other negative indicators of welfare. Moreover, these tool-use tasks can provide data on learning (Hirata and Celli 2003; Hirata and Morimura 2000; Paquette 1992), tool modification (Hopper et al. 2015), cognitive flexibility (Hopkins et al. 2014), physical reasoning (Brooks et al. 2021) and problem solving (Seed et al. 2009), or other characteristics such as manual laterality (McGrew and Marchant 1992; Sanz et al. 2016) and dexterity (Bardo et al. 2017; Osuna-Mascaró et al. 2021). It seems likely that enrichment devices that promote tool use will provide more cognitive stimulation than those that do not require tools. In fact, tool use, and more specifically flexible tool use (i.e., the ability to adapt to new situations through innovative solutions), is considered a complex activity that involves motivational, cognitive (i.e., information seeking and recombination) and sensorimotor aspects (i.e., manipulative propensity and specific manipulative skills) (Call 2013; Hunt et al. 2013).

Enrichment activities provided in a social setting might affect social dynamics within the group (Clark 2017), for example, by influencing affiliative or agonistic interactions. However, the few studies that have examined this have reported contradictory results, possibly due to methodological differences. For example, competition for access to the enrichment device may lead to aggression (Maki et al. 1989; Sha et al. 2012), although this is less likely if various subjects can simultaneously access the device (Brent and Eichberg 1991; Yamanashi et al. 2016). Similarly, affiliative interactions may also be positively or negatively affected by an enrichment (Brent and Eichberg 1991; Clark and Smith 2013; Sha et al. 2012), or not affected at all (Yamanashi et al. 2016).

Other aspects that should be considered when implementing a new enrichment procedure include subjects’ participation (i.e., engagement with the device, or proportion of time interacting with it) (Dutton et al. 2018; Lutz and Novak 2005; Schapiro and Lambeth 2007), as particularly in a social setting this might be affected by factors such as rank, personality, sex or age (Celli et al. 2003; Hopper et al. 2014). For instance, dominant chimpanzees may monopolize enrichment devices (Bloomstrand et al. 1986; Celli et al. 2003; Honess and Marin 2006; Paquette and Prescott 1988), which could negatively affect the acquisition of new tool-use behaviors by low-ranking individuals (Paquette 1992). Another important aspect is the level of difficulty of the task, which must be sufficiently stimulating to motivate the animals, yet solvable enough to avoid frustration (Meehan and Mench 2007). Currently there is no consensus on how to evaluate the level of cognitive stimulation and therefore the effectiveness of a particular cognitive enrichment (Clark 2017). Moreover, non-human primates can quickly become habituated to novel devices or tasks (Clark 2011; Vick et al. 2000), leading to the reduced effectiveness of enrichment activities over time (Tarou and Bashaw 2007). However, the effects of the enrichment may widely vary across subjects (Coleman and Novak 2017; Costa et al. 2018; Izzo et al. 2011). For example, Clark and Smith (2013) found that two out of six chimpanzees barely touched an enrichment device, whereas the others frequently interacted with it. Such variation highlights the importance of a more individual approach when evaluating the outcomes of a particular enrichment. This might include assessing subjects’ emotional states when interacting with an enrichment device, by measuring the occurrence of self-directed behaviors (e.g., scratching, touching or rubbing their body or face), which are reliable indicators of negative emotional states (i.e., stress or anxiety) in non-human primates (Baker and Aureli 1997; Maestripieri et al. 1992). Several studies have shown increases in self-directed behaviors in great apes faced with novel or more difficult tasks (Elder and Menzel 2001; Itakura 1993; Leavens et al. 2004, 2001; Meyer and Hamel 2014) or in response to errors (Leeds and Lukas 2018; Wagner et al. 2016; Yamanashi and Matsuzawa 2010). Furthermore, reported increases in self-directed behaviors in response to changes in non-human primates’ environments (Bonnie et al. 2016; Lukas et al. 2003) suggest that the simple presence of enrichment devices may also lead to such increases.

We evaluated the effects of a novel cognitive enrichment that requires tool use on solitary and social behaviors in two groups of sanctuary-housed chimpanzees. We hypothesized that the enrichment device would overall have a positive effect on the chimpanzees’ welfare by promoting species-typical behaviors and reducing negative ones, while also affecting social interactions. In particular, we predicted that the chimpanzees’ interest in the device (i.e., participation) would decrease across enrichment sessions (prediction 1), but that greater participation would be linked to an increase in tool use (prediction 2) and a reduction in negative indicators of welfare, such as abnormal behaviors (prediction 3) and inactivity (prediction 4). Moreover, we predicted that participation would increase social proximity (as the device could be used by more than one chimpanzee at a time; prediction 5), decrease affiliative behaviors (as chimpanzees would spend more time interacting with the enrichment and less time in grooming, social play or sexual behavior; prediction 6), and increase aggression-related behaviors (due to possible competition for the enrichment device; prediction 7). Finally, considering that our subjects had no previous experience with the enrichment device and the complexity of the task, we expected an increase in the occurrence of self-directed behaviors during engagement with the device, but not when simply in its presence (prediction 8).

## Materials and methods

### Subjects and study site

The study subjects were 14 adult chimpanzees (*Pan troglodytes*) living in two mixed-sex groups, each comprising seven individuals (Mutamba and Bilinga). The Mutamba group was composed of two females and five males, aged between 15 and 35 years (mean ± SD = 24.4 ± 8.2 years), and the Bilinga group was composed of three females and four males, aged between 17 and 36 years (mean ± SD = 29.1 ± 6.7 years). Both groups were housed at Fundació Mona, a center in Girona, Spain, dedicated to the rescue and rehabilitation of non-human primates that had been used as pets or in the entertainment industry. The chimpanzees spent their daytime hours in a 5640-m^2^ outdoor enclosure, divided into two areas (2420 m^2^ and 3220 m^2^), both containing natural vegetation and wooden platforms, towers, and ropes. Two observation huts around the perimeter allowed behavioral observation of both groups. The chimpanzees also had 140 m^2^ of indoor facilities where they spent the nights, but access to these was usually restricted during the daytime.

### Task description and experimental procedure

**Fig. 1:**
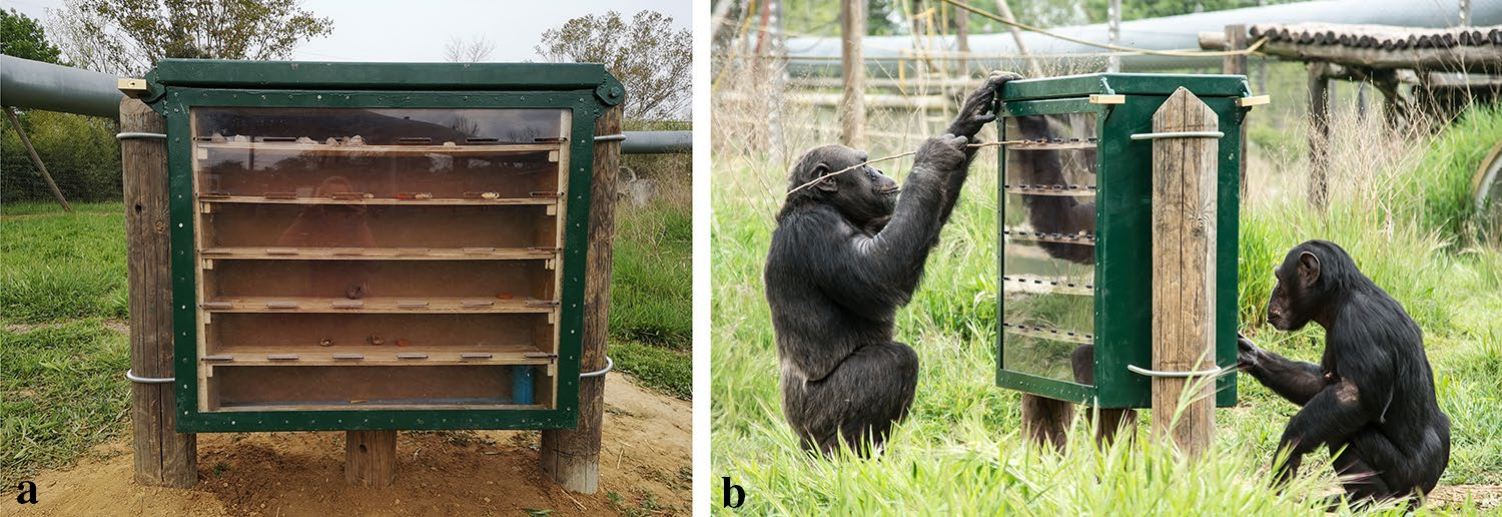
Frontal view of the double-sided food maze used in this study (**a**). Chimpanzees using tools to extract the food rewards from the maze (**b**). Photo credits: Miquel Llorente

The enrichment device was a double-sided maze consisting of a rectangular steel structure (approximately 1 × 0.5 m) with frontal transparent plastic panels and wooden shelves with holes at the ends (Fig. 1a). The maze could be filled with preferred food items (dried fruits and nuts), which the chimpanzees had to extract by using sticks or branches obtained from the natural vegetation in the outside enclosures (see Online Resource 1). No additional tools or materials were provided, but to facilitate learning and maintain the chimpanzees’ motivation, we randomly distributed food rewards on all the shelves of the device (rather than only on the upper shelf). Before filling the maze, the rewards were weighed and approximately the same quantity of food was removed from the chimpanzees’ midday snack to ensure a consistent daily caloric intake. Unlike similar food puzzles described in other studies, the device was double-sided, with two identical, independent mazes within the same structure, separated by an opaque middle panel (see Online Resource 1). Therefore, two chimpanzees could interact with the device at the same time, one on each side of the device (see Fig. 1b).

Each group of chimpanzees had one maze in the enclosure. The mazes were designed for this study, and were unfamiliar to the chimpanzees. Data collection started 1 week after the mazes were first installed in the enclosures, so that the chimpanzees could habituate to them. Then, for each group, we conducted behavioral observations on 24 randomly distributed days over a 2- to 3-month period (Mutamba group, 18 April–19 June 2019; Bilinga group, 17 June–12 September 2019): 12 enrichment days (with the loaded food maze), and 12 baseline days (with the empty maze). The mazes were filled in the morning, before the chimpanzees went into the outdoor enclosures, and were available to the chimpanzees throughout the daytime (approximately from 10.30 a.m to 6.00 p.m). No additional enrichment devices were provided on baseline and enrichment days, but during the rest of the study period other enrichments were sometimes provided in line with the usual routines of the center (e.g., bottles of juice, baited fabrics, hoses filled with food).

### Behavioral observations

On baseline and enrichment days we collected behavioral data for a total of 2 h 40 min/day, divided into two 80-min sessions, one in the morning during the period from 10.30 a.m to 2.00 p.m, and one in the afternoon during the period from 3.00 p.m to 6.00 p.m. We expected the chimpanzees to use the maze more frequently in the morning, when it became available. Given the novelty and complexity of the task, however, we also expected that some rewards would not be extracted right away, and so we also collected data in the afternoon. No observations were conducted around midday, as this was the chimpanzees’ feeding time and usually corresponded to very low activity levels in the group.

We collected data using instantaneous scan sampling (every 2 min) and untimed-event focal sampling (10 min per subject) (Bakeman and Quera 2011). Scan sampling allowed us to record mid- to long-duration behaviors including (1) engagement with the enrichment, (2) tool use, (3) abnormal behaviors, (4) inactivity, (5) social proximity, (6) affiliation-related behaviors, (7) aggression-related behaviors. Descriptions of the behaviors can be found in Table 1. As some behaviors were not mutually exclusive, in each scan sample interval a chimpanzee could exhibit more than one behavior at the same time (see details in Table 1). Total scan sampling observation time was 128 h, equally distributed between conditions and groups, i.e., 960 scans per condition and group. The untimed-event focal sampling focused on rare or short-duration behaviors (e.g., self-directed behaviors). Based on the definitions in the literature (Leavens et al. 2001; Schino et al. 1996; Yamanashi and Matsuzawa 2010), self-directed behaviors included rubbing and scratching directed towards the face and body (see detailed definition in Table 1), as these have been consistently linked to stress or anxiety, but we excluded self-grooming because it may not always be a good proxy for stress (Meyer and Hamel 2014). Following previous studies (Hopkins et al. 2006; Wagner et al. 2016), the incidence of self-directed behaviors was measured as the number of bouts. A bout ended when (1) it stopped for 3 s or more, (2) limb to body contact ceased, or (3) the body target changed. Focal observations were conducted in a pseudo-randomized order, the aim of which was to observe each chimpanzee for at least 10 min in the morning and in the afternoon on each day. If no data were collected because a chimpanzee was not present in the outdoor enclosures during an observation session, we conducted an additional 10-min observation in a later session. Due to observer absence, for one chimpanzee group we conducted focal observations on only 10 of the 12 baseline days; therefore, we randomly selected 10 data collection days for each condition and group for the analysis of self-directed behaviors. Therefore, each chimpanzee was observed for a total of 3.3 h (200 min) in each condition (baseline and enrichment).

**Table 1:** Behavioral catalogue

Behavioral category	Definition
1. Participation	The chimpanzee is actively interacting with or in contact with the food maze while exploring it with the hands, feet or mouth
2. Tool use^a^	The chimpanzee uses a mobile element, external to the body (the tool), to perform a goal-oriented action on another element that modifies its physical properties. It includes tool modification and tool transportation
3. Abnormal behaviors	The chimpanzee displays maladjusted stereotypical behaviors such as rocking, pacing, self–harm, coprophagy (eating feces), regurgitation, re-ingestion, trichotillomania (hair-pulling), trichotillophagia (hair-pulling and eating hair), ear-poking, eye-poking
4. Inactivity	The chimpanzee does not perform any action or activity other than sitting or lying down. It includes self-inspection, yawning, and sleeping
5. Social proximity^b^	The chimpanzee is at less than one-arm’s length from one or more subjects while performing a solitary activity, with no social interaction between subjects
6. Affiliation-related behaviors	The chimpanzee exhibits one of the following behaviors: (1) grooming—body-cleansing of one individual by another (includes mutual grooming), performed using the fingers or the mouth; (2) social play—playful behavior between two or more individuals associated with behavioral indicators of play (e.g., play face, laughter, friendly head bobbing, softly knocking on the ground, playful chasing); (3) sexual behavior—sexual interaction, or search for sexual interaction, between two individuals, including behaviors such as copulation, attempted copulation, genital presentation and other behaviors directed towards the genitals of another individual; (4) other behaviors identified as affiliative, but not fitting the criteria of grooming, social play or sexual activity (e.g., embracing, co-feeding, following)
7. Aggression-related behaviors	The chimpanzee exhibits one of the following behaviors: (1) agonistic dominance—threat-related behaviors such as direct aggression, charging display, displacement and resource appropriation (e.g., stealing food or objects) (the behavior may be accompanied by vocalizations); (2) agonistic submission—avoidance, teeth baring, display, food submission (e.g., leaving/dropping food and moving away when others try to steal it), hand-to-mouth, finger-to-mouth (the behavior may be accompanied by vocalizations such as panting/grunting, and includes running away from others in conflict situations); (3) other behaviors occurring in agonistic contexts, but not fitting the criteria of agonistic dominance or agonistic submission (e.g., appeasement, consolation, reconciliation, and requesting support)
8. Self-directed behaviors	The chimpanzee displays one of the following behaviors: (1) scratching—continuous movement of the hand over the skin involving the ends of the digits or nails; (2) rubbing—continuous movement of the hand over the skin not involving the ends of the digits, performed either with the palmar, dorsal or lateral side of the hand [this category also includes self-touching such as nose wiping (Yamanashi and Matsuzawa 2010) or face stroking (Itakura 1993)]
Not visible/not present	The chimpanzee or the behavior cannot be identified, or the chimpanzee is not in the outdoor enclosure (e.g., he/she is in the sleeping area or in an outdoor cage)

Note: data on all behaviors were collected using 2-min instantaneous scan sampling, except for those on self-directed behaviors, which were collected using untimed-event focal sampling

^a^Tool use could occur simultaneously with participation

^b^Social proximity and solitary behaviors (behaviors 1–4) were also not mutually exclusive (i.e., individuals could be in social proximity while simultaneously engaging in one of these behaviors)

To further investigate the effect of the food maze on self-directed behaviors, we videotaped every enrichment session and later coded the data for self-directed behaviors when chimpanzees interacted with the task. For consistency in observation time for baseline and enrichment sessions (with no task interaction), we coded data for approximately 20 min/day per subject when they were interacting with the enrichment (i.e., 10 min in the morning and 10 min in the afternoon). If the duration of subject participation was less than 10 min, we repeated the observation when the chimpanzee resumed participation, and so on until we reached a total observation time of 10 min. To maximize the amount of data on self-directed behaviors while manipulating the enrichment, we videotaped for an additional 30 min per session, and used the video recordings of all 12 enrichment sessions. However, it was not possible to reach 3.3 h of total observation time per subject as in the baseline and enrichment conditions (with no task interaction), as most chimpanzees interacted with the food maze for less time over the whole study period.

### Inter-observer reliability

Observations were conducted by several researchers, who had completed a period of training and had to pass an inter-observer reliability test (agreement between observers ≥ 85%) before collecting data. All the data were collected using ZooMonitor (Ross et al. 2016), an application which facilitates the recording and analysis of animal behavior (Wark et al. 2019).

### Data analysis

To investigate chimpanzee use of the enrichment device over time and assess the effect of participation on behavior, we ran eight different generalized linear mixed models (GLMM) (Baayen 2008) using the glmmTMB package (Brooks et al. 2017) in R. Model 1 assessed whether participation during enrichment (i.e., the number of scans involving interaction with the device in an enrichment session out of all the scans for that individual) varied across time, and whether individual characteristics—such as sex and age—predicted participation. In this model, we entered one line per individual and session (only including enrichment sessions), with session number, sex, age and time of the day (morning vs. afternoon) as test predictors. We further included group as the control and subject identity as a random effect, using a beta distribution.

We then assessed whether participation (operationalized as in model 1) predicted the occurrence of Tool use (model 2), Abnormal behaviors (model 3), Inactivity (model 4), Social proximity (model 5), Affiliation-related (model 6) and Aggression-related behaviors (model 7), and whether the effects varied across sessions (models 2–7). In all these models we included one line per subject and session. The dependent variables (i.e., the behaviors given above) were operationalized as the number of scans in which the subject performed the behavior divided by the total number of scans in which the subject was visible. Being proportions, these variables were modeled with a beta distribution. Then, we entered as test predictors the two-way interactions between participation and session number, and their main effects. If we detected overdispersion (models 3 and 7), we re-ran the models after transforming response and participation into binomial variables using a binomial distribution. No overdispersion was detected in the models presented below. Finally, model 8 assessed whether Self-directed behaviors were affected by the enrichment. In this model, we also included one line per subject and session, and we operationalized the dependent variable (i.e., self-directed behaviors) as the total number of bouts performed in the time the subject was visible. This variable was a count and, to avoid overdispersion, it was modeled with a negative binomial distribution, adding observation time as an offset in the model. In model 8, the test predictors were the two-way interactions (and their main effects) between session number and the categorical predictor Condition (i.e., whether the observation was conducted during the Baseline, when the enrichment was not present; during the Enrichment No Interaction, when the enrichment was present but the subject was not interacting with it; or during the Enrichment Interaction, when the enrichment was present and the subject was manipulating it). In models 2–8, we entered sex, age, group and time of the day (morning, evening) as control predictors, with subject identity included as a random effect.

In all of the models, age was* z*-transformed to facilitate model convergence. To compare full models containing all predictors with null models containing only controls and random factors, we used a likelihood ratio test (function anova) (Dobson 2002) and a significance level of 0.05. If the full model significantly differed from the null model, we obtained the *p*-values for each test predictor via single-term deletion using the R function drop1 (Barr et al. 2013). If the two-way interactions (which always included their main effects) were not significant, we downgraded them and re-ran the models including only main effects. In the case of significant categorical predictors with more than two levels (model 8), we used Tukey tests in the emmeans package (Lenth 2020) to compare the different levels. To rule out collinearity, we calculated variance inflation factors (Field 2009), which were very low for all of the models (maximum variance inflation factors across all models = 1.34).

## Results

Participation in the enrichment varied widely across individuals (mean ± SD = 8.92 ± 15.27% scans, range = 0.22–53.52%), with all the chimpanzees interacting with the device, but some only very briefly (< 1% of scans). One female (Africa; Mutamba group) was particularly interested in the maze, and spent more than 50% of scans engaged with it in the enrichment condition. Two other females (Coco, Bilinga group; Waty, Mutamba group), also spent a high proportion of scans interacting with the device (around 30% and 15%, respectively). Only two of these three females (Africa and Coco) mastered the task, reliably retrieving the rewards from the maze. The other chimpanzees interacted with the maze, usually with tools, but they did not succeed in moving the rewards across the different shelves of the maze. Tables S1 and S2 show individual and mean values of participation and the incidence of other behaviors in the baseline and enrichment conditions. Tables 2 and 3 show a summary of the predictions and results for models 1–8.

**Table 2:** Summary of predictions and results for models 1–8

Predictions	Supported?	Model
1. Participation in the enrichment—		1
Remains constant across sessions	No	
Is affected by sex	Yes	
Is affected by age	No	
Is affected by: time (morning/afternoon)	Yes	
Participation in the enrichment predicts a consistent—		
2. Increase in tool use	Yes	2
3. Decrease in abnormal behaviors	No	3
4. Decrease in inactivity	Yes	4
5. Increase in social proximity	No	5
6. Decrease in affiliation-related behaviors	No	6
7. Increase in aggression-related behaviors	Yes^a^	7
8. Interaction with the enrichment predicts an increase in the rate of self-directed behaviors compared to the baseline and enrichment conditions	Yes	8

^a^In model 7, participation predicted an increase in aggression-related behaviors over time (i.e., across sessions)

**Table 3:** Estimates, SE, confidence intervals (*CI*), likelihood ratio tests (*LRT*),* df* and *p*-values for all variables in models 1–8 (the reference category is given* in parentheses*)

Models	Estimate	SE	CI (2.5%)	CI (97.5%)	LRT	*df*	*p*
Model 1: Participation							
Intercept	− 1.60	0.32	–	–	–	–	–
Session number	− 0.04	0.02	− 0.07	− 0.01	5.395	1	*0.020*
Sex (male)	− 1.05	0.35	− 1.74	− 0.36	6.873	1	*0.006*
Age	− 0.24	0.18	− 0.59	0.10	1.758	1	0.185
Time (afternoon)	− 0.67	0.12	− 0.90	− 0.44	32.251	1	* < 0.001*
Group (Mutamba)^a^	0.50	0.35	− 0.19	1.19	1.898	1	0.168
Model 2: Tool use							
Intercept	− 2.81	0.18	–	–	–	–	–
Participation	1.05	0.10	0.85	1.25	84.129	1	* < 0.001*
Session number	− 0.01	0.01	− 0.03	0.01	1.060	1	0.303
Sex (male)^a^	− 0.41	0.17	− 0.73	− 0.08	4.994	1	*0.025*
Age^a^	− 0.07	0.08	− 0.24	0.09	0.732	1	0.392
Time (afternoon)^a^	− 0.14	0.08	− 0.29	0.02	2.969	1	0.085
Group (Mutamba)^a^	− 0.05	0.17	− 0.38	0.28	0.089	1	0.765
Model 3: Abnormal behaviors							
Intercept	− 2.75	0.87	–	–	–	–	–
Participation	− 0.77	0.78	− 2.31	0.76	–	–	–
Session number	− 0.03	0.04	− 0.12	0.05	–	–	–
Sex (male)^a^	− 0.05	0.95	− 1.92	1.82	0.003	1	0.958
Age^a^	0.15	0.50	− 0.83	1.13	0.090	1	0.764
Time (afternoon)^a^	0.52	0.29	− 0.05	1.08	3.286	1	0.072
Group (Mutamba)^a^	− 0.14	0.97	− 2.05	1.77	0.0215	1	0.883
Participation × session number	0.03	0.11	− 0.19	0.25	0.0697	1	0.792
Model 4: Inactivity							
Intercept	0.13	0.28	–	–	–	–	–
Participation	− 1.98	0.35	− 2.67	− 1.29	34.092	1	* < 0.001*
Session number	0.01	0.01	− 0.01	0.04	1.140	1	0.286
Sex (male)^a^	− 0.21	0.31	− 0.82	0.40	0.439	1	0.508
Age^a^	0.11	0.16	− 0.19	0.42	0.521	1	0.471
Time (afternoon)^a^	− 0.44	0.08	− 0.61	− 0.28	27.179	1	* < 0.001*
Group (Mutamba)^a^	− 0.50	0.31	− 1.11	0.11	2.364	1	0.124
Model 5: Social proximity							
Intercept	− 1.53	0.19	–	–	–	–	–
Participation	− 0.34	0.32	− 0.97	0.28	1.176	1	0.278
Session number	− 0.03	0.01	− 0.05	− 0.01	6.569	1	*0.010*
Sex (male)^a^	− 0.62	0.19	− 1.00	− 0.24	7.780	1	*0.005*
Age^a^	0.01	0.10	− 0.17	0.20	0.024	1	0.878
Time (afternoon)^a^	− 0.48	0.08	− 0.64	− 0.32	33.761	1	* < 0.001*
Group (Mutamba)^a^	− 0.06	0.19	− 0.43	0.32	0.091	1	0.763
Model 6: Affiliation-related behaviors							
Intercept	− 2.31	0.18	–	–	–	–	–
Participation	− 0.47	0.61	− 1.67	0.72	–	–	–
Session number	0.00	0.01	− 0.02	0.03	–	–	–
Sex (male)^a^	− 0.48	0.16	− 0.79	− 0.17	6.967	1	*0.008*
Age^a^	0.04	0.08	− 0.11	0.20	0.319	1	0.572
Time (afternoon)^a^	0.24	0.08	0.08	0.41	8.347	1	*0.004*
Group (Mutamba)^a^	0.39	0.16	0.08	0.70	4.970	1	*0.026*
Participation × session number	0.01	0.08	− 0.15	0.17	0.008	1	0.928
Model 7: Aggression-related behaviors							
Intercept	− 4.33	0.77	–	–	–	–	–
Participation	− 1.93	0.87	− 3.64	− 0.22	–	–	–
Session number	− 0.14	0.05	− 0.24	− 0.05	–	–	–
Sex (male)^a^	2.22	0.67	0.90	3.54	9.534	1	*0.002*
Age^a^	− 0.37	0.25	− 0.87	0.13	1.922	1	0.166
Time (afternoon)^a^	0.69	0.30	0.10	1.28	5.369	1	*0.020*
Group (Mutamba)^a^	1.07	0.56	− 0.03	2.17	3.576	1	0.059
Participation × session number	0.29	0.12	0.06	0.52	5.890	1	*0.015*
Model 8: Self-directed behaviors							
Intercept	0.56	0.17	–	–	–	–	–
Condition (Baseline)	− 0.70	0.10	− 0.89	− 0.51	75.138	1	* < 0.001*
Condition (Enrichment no interaction)	− 0.85	0.10	− 1.04	− 0.65			
Session number	− 0.02	0.01	− 0.04	0.00	2.460	1	0.117
Sex (male)^a^	0.20	0.16	− 0.12	0.53	1.462	1	0.227
Age^a^	0.08	0.08	− 0.08	0.24	1.031	1	0.310
Time (afternoon)^a^	− 0.20	0.07	− 0.33	− 0.06	7.678	1	*0.006*
Group (Mutamba)^a^	− 0.42	0.16	− 0.74	− 0.10	5.359	1	*0.021*

^a^Control variables

### Participation (model 1)

**Fig. 2:**
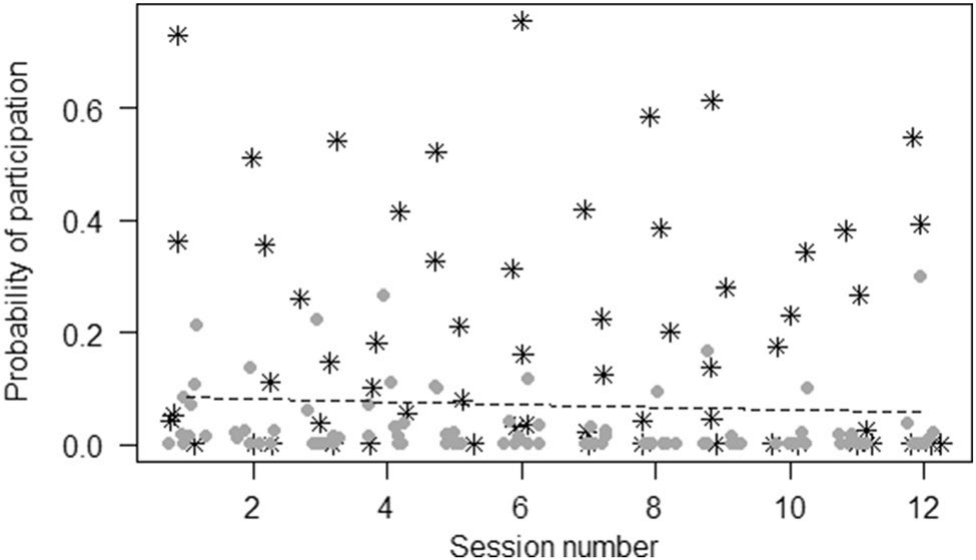
Jitter plot showing probability of participating in the enrichment activity as a function of session number.* Asterisks* represent female chimpanzees and* circles* male chimpanzees in each session. The* dashed line* represents the fitted model, which is like model 1 but unconditional on all the other predictors that were standardized

For model 1, the full model significantly differed from the null model (GLMM, *χ*^2^ = 44.41,* df* = 4, *p* < 0.001). Participation decreased across sessions (*p* < 0.05) (Fig. 2), and was higher in the morning than in the afternoon (*p* < 0.001). Females participated significantly more than males (*p* < 0.05), but age did not significantly predict participation.

### Solitary and social behaviors (models 2–9)

**Fig. 3:**
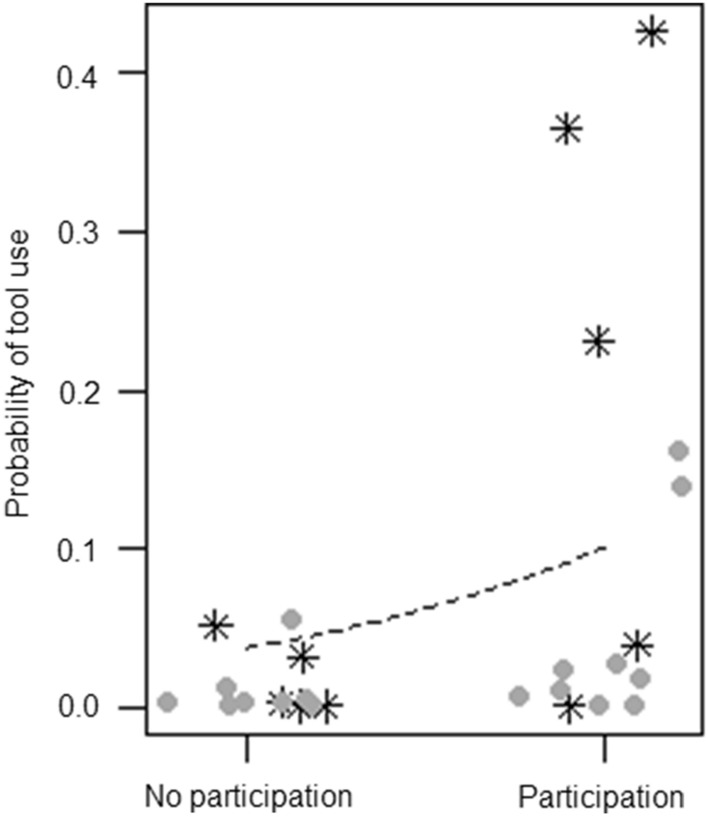
Jitter plot showing probability of using tools as a function of whether individuals participated in the enrichment activity.* Asterisks* represent female chimpanzees and* circles* male chimpanzees in the two conditions. The* dashed line* represents the fitted model, which is like model 2 but unconditional on all the other predictors that were standardized

**Fig. 4:**
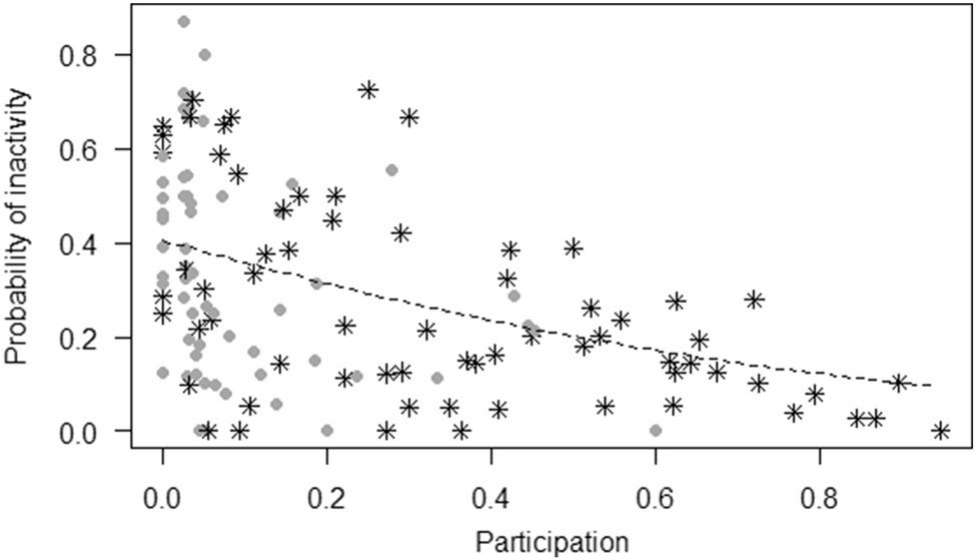
Jitter plot showing probability of being inactive as a function of participation in the enrichment activity.* Asterisks* represent female chimpanzees and* circles* male chimpanzees in each session. The* dashed line* represents the fitted model, which is like model 4 but unconditional on all the other predictors that were standardized

**Fig. 5:**
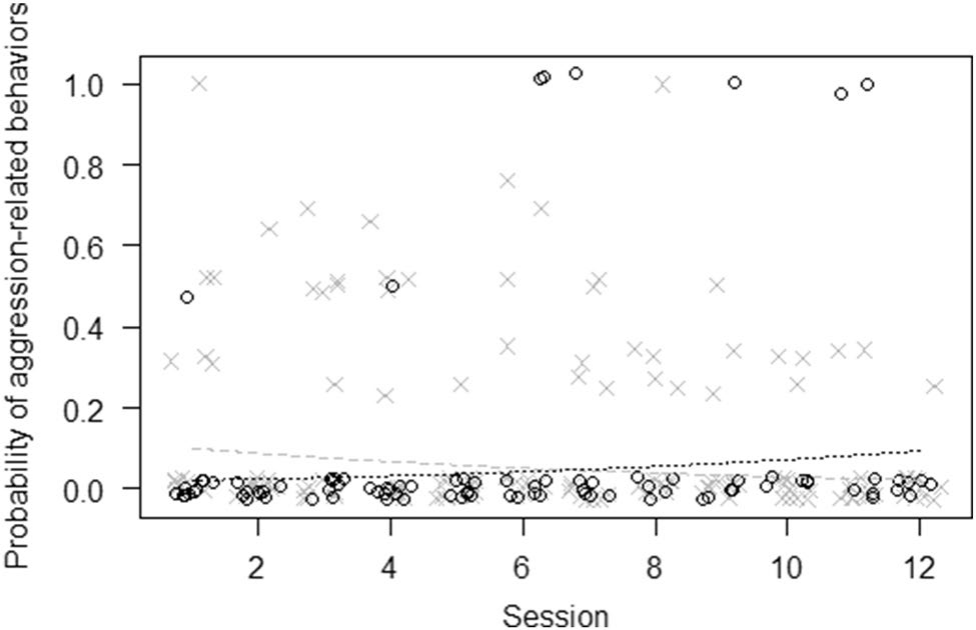
Jitter plot showing probability of showing aggression-related behaviors as a function of session number and separately for individuals who participated and for those who did not participate in the enrichment activities.* Circles* represent chimpanzees that participated in the enrichment activity in each session and* crosses* those that did not participate. The* dashed line* represents the fitted model, which is like model 7 but unconditional on all the other predictors that were standardized

For model 2 (tool use), the full–null model comparison was significant (GLMM, *χ*^2^ = 87.05,* df* = 3, *p* < 0.001), revealing that participation in the enrichment increased tool use (*p* < 0.001), with no differences across sessions (Fig. 3). For model 3 (abnormal behaviors), the full–null model comparison was not significant (GLMM, *χ*^2^ = 2.56,* df* = 3, *p* = 0.464), whereas for model 4 (inactivity), the full model significantly differed from the null model (GLMM, *χ*^2^ = 35.93,* df* = 3, *p* < 0.001), revealing that participation in the enrichment was linked to a decrease in inactivity (*p* < 0.001), with no differences across sessions (Fig. 4). For model 5 (social proximity), the full–null model comparison was significant (GLMM, *χ*^2^ = 7.99,* df* = 3, *p* < 0.05), but participation had no effect on social proximity (*p* = 0.278), which decreased across sessions (*p* = 0.010). For model 6 (affiliation-related behaviors), the full–null model comparison was not significant (GLMM, *χ*^2^ = 2.01,* df* = 3, *p* = 0.569), whereas for model 7 (aggression-related behaviors) the full model significantly differed from the null model (GLMM, *χ*^2^ = 11.72,* df* = 3, *p* < 0.05), showing that only individuals participating in the enrichment increased aggression-related behaviors across sessions (*p* < 0.05) (Fig. 5).

### Self-directed behaviors (model 8)

For model 8, the full–null model comparison was significant (GLMM, *χ*^2^ = 80.23,* df* = 5, *p* < 0.001), revealing a significant increase in self-directed behaviors when chimpanzees interacted with the enrichment as compared to when they did not (i.e., in the baseline condition and in the enrichment condition without task interaction, both *p* < 0.001).

## Discussion

The aim of this study was to assess the effects of a novel tool-based cognitive feeding enrichment on solitary and social behaviors of sanctuary-housed chimpanzees. We found that engagement with the enrichment decreased across sessions, strongly varied across subjects and was higher in females. As expected, participation was linked to an increase in tool use, a decrease in inactivity, and an increase in agonistic behaviors. However, in contrast to our predictions, participation had no effect on abnormal behaviors, social proximity or affiliative behaviors. Finally, we detected increased self-directed behaviors when subjects interacted with the enrichment device, as compared to when they did not in either baseline or enrichment sessions.

Among our subjects, three females (Africa, Coco and Waty) were the most frequent users of the enrichment, spending between 15 and 50% of the scans interacting with the food maze. The other chimpanzees participated much less, with some hardly interacting with the device at all. Wide inter-individual variation in participation has been reported in other studies that presented cognitive devices to great apes (Clark et al. 2019; Clark and Smith 2013; Tarou et al. 2004) and monkeys (Jacobson et al. 2019; Polgár et al. 2017). It is noteworthy that only Africa and Coco, two of the three more frequent users of the food maze, were able to master the task when the food rewards were in the upper levels of the maze, by moving them across all of the vertical levels. When chimpanzees used a puzzle board containing food rewards in a study by Brent and Eichberg (1991), females also used the device more often than males. Similarly, Yamanishi et al. (2016) found that female chimpanzees mastered new tool-using behaviors faster than males. Therefore, our results are consistent with previous studies supporting sex differences in captive chimpanzees’ tool use and proficiency, a pattern that has been repeatedly observed in the wild (Boesch and Boesch 1981, 1990; Lonsdorf 2005; Lonsdorf et al. 2004; McGrew 1979; Pruetz et al. 2015), and in both captive (Boose et al. 2013; Gruber et al. 2010) and wild bonobos (Samuni et al. 2022). Considering our small sample size, however, our findings regarding sex differences should be interpreted with caution.

In line with our predictions, participation decreased across sessions, as observed in other studies in which non-human primates lost interest in puzzle-feeders within several hours of their exposure to them (Bloomstrand et al. 1986; Csatádi et al. 2008). Indeed, non-human primates can quickly become habituated to various novel enrichment devices or tasks (Paquette 1992; Vick et al. 2000). Nonetheless, the level of difficulty should be taken into account when assessing subjects’ interest in the enrichment, as complex puzzle feeders might promote subjects’ long-term engagement (Clark 2011; Taylor et al. 1994). As only two of our chimpanzees were able to extract the rewards from the maze, the task was clearly not that easy. The decrease in participation over time was likely due to almost all of the chimpanzees approaching and trying to solve the maze at first, but then giving up after several failed attempts (especially for rewards on the upper levels of the maze). Thus, failure to master the task might have led to frustration and loss of motivation (Toates 1986). Our chimpanzees had previous experience with other tool-based enrichments, such as artificial termite mounds, from which they successfully retrieved food rewards (Padrell et al. 2021). These tasks also involved searching for and modifying tools from the environment, but dipping to extract food appears to be less complex than guiding food rewards through a maze, which requires fine motoric skills, precise hand movements, and probably higher cognitive abilities such as planning or an understanding of an object’s physical properties (Völter and Call 2014). Furthermore, wild primates can take years to fully master tool-based activities like ant-dipping or nut-cracking (Boesch and Boesch-Achermann 2000; Matsuzawa et al. 2001; Ottoni and Izar 2008). Thus, the chimpanzees in our study, with no prior experience of this type of device, might have needed more time and practice to master the maze.

Overall, our results reveal the importance of considering individual differences when implementing enrichment activities (Coleman and Novak 2017). Variables like sex, age, cognitive skills and personality may strongly affect how subjects respond to a particular cognitive challenge (Altschul et al. 2017; Herrelko et al. 2012; Hopper et al. 2014) and contribute to large differences in participation and success in extracting food from enrichment devices. Additionally, although we used highly preferred food rewards, variability in the subjects’ food preferences or food motivation might also have affected participation. Other factors that should be taken into consideration include past experiences and rearing conditions (e.g., Brent et al. 1995; Gluck et al. 1973; Morimura and Mori 2010; Novak and Sackett 2006; Simpson et al. 2019). Unfortunately, however, reliable and precise information about the past life of a rescued chimpanzee is usually unavailable. Finally, it should also be noted that, due to the limited number of agonistic interactions and low rank stability in our chimpanzee groups, we did not include rank in our analyses; future studies on larger groups with stable hierarchies should consider the possible effects of rank on enrichment-related activities.

As expected, and as previously reported in other studies involving puzzle feeders, participation was related to an increase in tool use and a reduction of inactivity, while promoting feeding (Brent and Eichberg 1991; Csatádi et al. 2008; Gilloux et al. 1992; Roberts et al. 1999). However, in contrast to our predictions and the results of some other studies that used puzzle feeders, enrichment was not linked to a reduction in abnormal behaviors in our chimpanzees (see Brent and Eichberg 1991; Maki et al. 1989; Yamanashi et al. 2016). In fact, in our sample, abnormal behaviors were already infrequent (fewer than 1% of the scans in the baseline condition; see Table S1), compared to the 2.9–7.6% of time spent in abnormal behaviors reported for captive chimpanzees in other studies (Bradshaw et al. 2008). Furthermore, abnormal behavior may to some degree be endemic in captive populations (Birkett and Newton-Fisher 2011), and very difficult to eradicate in subjects that have experienced trauma in the past (Lopresti-Goodman et al. 2012), which is the case for some of our chimpanzees.

Considering its novelty, we expected the chimpanzees to gather around the device to explore it and possibly to observe others performing the task. Additionally, the device contained two simultaneously available but independent mazes, usable by two chimpanzees at the same time without mutual interference. However, contrary to our predictions, we found no increase in social proximity for those who participated more, as the maze was usually monopolized by a single chimpanzee in each group (typically one of the females who learned to retrieve the rewards). We further predicted that chimpanzees who participated more would show a decrease in affiliative behaviors due to spending more time at the maze and therefore investing less time in social interactions. In contrast to previous studies (e.g., Brent and Eichberg 1991), however, interacting with the enrichment did not disrupt the occurrence of usual social activities. Thus, our results are in line with those reported by Yamanashi et al. (2016) and Padrell et al. (2021), who also found no changes in the occurrence of affiliative behaviors resulting from tool-based enrichments. Nonetheless, we did find a positive association between participation and agonistic behaviors, which increased across sessions. Although the food maze could be used by more than one chimpanzee at a time, it appeared to promote competition and thus increased aggression (Jacobson et al. 2019; Maki et al. 1989), as expected when tasks are presented in a social setting (Tarou et al. 2004). This may be especially important in our group, considering that all the chimpanzees who failed to master the task were males, who are often aggressive towards females. Furthermore, it has been reported that wild female chimpanzees also tend to be aggressive in the context of feeding competition (Muller and Mitani 2005). One alternative to our method would have been to install single-maze devices (rather than double-sided mazes), in different areas of the enclosure (out of full view of other group members), to decrease direct competition. It should also be noted that, in our behavioral catalogue, agonistic behaviors included both aggressive and submissive behaviors, which are not necessarily indicators of poor welfare. Therefore, although aggression is not desirable in captive primates, the increase in aggression observed in our study may not have been a particularly negative outcome.

Interacting with the enrichment device was linked to an increase in self-rubbing and scratching, as compared to when no enrichment was present (baseline) or when it was present but the subject did not interact with it. These results reflect the complex relationship between enrichment and self-directed behaviors. Although enrichment is supposed to reduce stress-related behaviors, cognitive challenges are expected to trigger them, as a result of emotional arousal (Baker and Aureli 1997; Maestripieri et al. 1992). Thus, in our study, the increase in self-directed behaviors may not be an indicator of stress or anxiety, but rather an expression of arousal in a challenging context. Other studies involving tool-based tasks in social settings have also reported complex results regarding self-directed behaviors. For instance, Yamanashi et al. (2016) found a decrease in self-directed behaviors when tool-based feeders were provided compared to when the enrichment was absent. By contrast, Clark and Smith (2013) found that in the presence of a cognitive device chimpanzees scratched themselves more, whereas using the device was associated with a decrease in scratching. Furthermore, a novel cognitive task presented to zoo-housed chimpanzees by Herrelko et al. (2012) caused no increase in self-directed behaviors (i.e., rubbing and scratching) during training, as compared to a baseline condition. However, in contrast to Herrelko et al. (2012), the chimpanzees in our study were observed in their usual enclosures, with the other group members continuously present. This might have increased competition for food, frustration, and agonistic behaviors (as we found). If the individuals had been observed with exclusive access to the device and no disturbance by other chimpanzees, their anxiety levels might have been lower. Nonetheless, providing these types of activities in a social context better simulates the natural conditions of chimpanzees, including intragroup competition, and thus increases ecological validity (Cronin 2017).

Environmental enrichment usually involves introducing novel stimuli with the ultimate goal of improving captive animal welfare (Azevedo et al. 2007; Sheperdson 2003; Young 2003). In this respect, the food maze in this study had a positive impact on chimpanzees’ behavior by (1) promoting tool use, which is a species-typical behavior that rarely occurs in captivity in the absence of specific enrichments; and (2) decreasing inactivity, which is usually considered a positive outcome of environmental enrichment for captive great apes (Baker 1997; Brent 1992; Brent and Eichberg 1991; Celli et al. 2003; Csatádi et al. 2008; Gilloux et al. 1992). Arousal levels, assessed through self-directed behaviors, were not affected by the presence of the enrichment device, but did increase for individuals interacting with it. Finally, one of our aims was to promote activity that stimulated the chimpanzees cognitively by creating learning opportunities that simulate the natural environment (Young et al. 2020), in which animals face challenging situations (e.g., finding food) that often require complex behavioral and cognitive skills such as exploration or problem solving (Shettleworth 2010). The food maze indeed presented a challenge, but as most of the chimpanzees failed to master the task during the study period, longer exposure might lead to better assessment of the impact of this and other similar enrichments on chimpanzee behavior.

## Supplementary Information

Below is the link to the electronic supplementary material.

Supplementary file1 Tables S1 and S2: Incidence of behaviors in the baseline (S1) and enrichment (S2) conditions. (DOCX 21 KB)

Supplementary file2 (TXT 85 KB)

Supplementary file3 (TXT 45 KB)

Supplementary file4 Online Resource 1: Video | Details of the structure and functioning of the food maze. (MP4 156873 KB)

## Data Availability

The data presented in this study are provided as Supplementary files (Supplementary files 2 and 3).
