# Supplementary material for: Cognitive enrichment in a social setting: assessing the use of a novel food maze in sanctuary-housed chimpanzees
Source: Primates. 2022 Jul 18;63(5):509–24. doi: 10.1007/s10329-022-00996-0 (PMC9463267; doi:10.1007/s10329-022-00996-0)
Supplement: Supplementary file 1 — Supplementary file1 Tables S1 and S2: Incidence of behaviors in the baseline (S1) and enrichment (S2) conditions. (DOCX 21 KB) [file 10329_2022_996_MOESM1_ESM.docx]

**Tables S1 and S2:** **Incidence of behaviors in the baseline (S1) and enrichment (S2) conditions.** Behaviors collected with scan sampling method (i.e., participation, tool use, inactivity, abnormal behaviors, social proximity, aggression-related and affiliation-related behaviors) represent the percentage of scans in which the behavior was observed), whereas behaviors collected with all-occurrence focal sampling (i.e., self-directed behaviors: rubs and scratches) correspond to rates (i.e., number of self-directed behaviors/ observation time in minutes).

**Table S1. Baseline condition**

| **Scan sampling** | | | | | | | | | | **Focal sampling** | |
| --- | --- | --- | --- | --- | --- | --- | --- | --- | --- | --- | --- |
| **Subject** | **Tool**  **use** | **Inactivity** | | **Abnormal**  **behaviors** | | **Social**  **proximity** | **Aggression-related**  **behaviors** | | **Affiliation-related**  **behaviors** | **Self-directed**  **behaviors** | |
| Africa | 0.24 | 64.03 | 0.72 | | 17.38 | | | 0.24 | 4.92 | 0.49 |  |
| Bea | 0.00 | 72.25 | 0.27 | | 23.44 | | | 0.00 | 9.12 | 0.61 |  |
| Bongo | 0.00 | 43.69 | 0.00 | | 3.63 | | | 3.38 | 2.46 | 0.57 |  |
| Charly | 0.00 | 10.09 | 3.29 | | 2.27 | | | 1.88 | 15.02 | 0.56 |  |
| Cheeta | 0.00 | 65.81 | 0.00 | | 20.16 | | | 0.00 | 4.88 | 0.73 |  |
| Coco | 5.61 | 38.14 | 3.37 | | 9.83 | | | 0.00 | 12.34 | 0.45 |  |
| Juanito | 0.17 | 41.01 | 0.34 | | 19.11 | | | 0.84 | 15.63 | 0.24 |  |
| Marco | 0.15 | 33.80 | 0.00 | | 4.80 | | | 1.23 | 6.02 | 0.50 |  |
| Nico | 0.85 | 45.96 | 0.00 | | 1.80 | | | 2.55 | 7.66 | 1.46 |  |
| Tico | 1.86 | 54.97 | 3.06 | | 3.94 | | | 0.00 | 0.00 | 0.92 |  |
| Tom | 0.54 | 55.09 | 0.27 | | 6.74 | | | 0.54 | 8.85 | 0.75 |  |
| Toni | 0.23 | 51.04 | 0.00 | | 0.16 | | | 0.69 | 6.24 | 0.43 |  |
| Victor | 3.36 | 56.32 | 2.42 | | 9.48 | | | 0.13 | 2.28 | 0.56 |  |
| Waty | 1.87 | 26.67 | 0.00 | | 12.50 | | | 0.53 | 37.07 | 0.33 |  |
| **Mean±SD** | **1.06±1.64** | **47.06±16.62** | | **0.98±1.38** | | **9.66±7.67** | **0.86±1.06** | | **9.46±9.19** | **0.62±0.30** | |

**Table S2. Enrichment condition**

| **Scan sampling** | | | | | | | | **Focal sampling** |
| --- | --- | --- | --- | --- | --- | --- | --- | --- |
| **Subject** | **Participation** | **Tool use** | **Inactivity** | **Abnormal**  **behaviors** | **Social**  **proximity** | **Aggression-related**  **behaviors** | **Affiliation-related**  **behaviors** | **Self-directed**  **behaviors** |
| Africa | 53.52 | 46.35 | 25.09 | 0.25 | 8.81 | 0.12 | 4.70 | 0.70 |
| Bea | 1.09 | 0.00 | 64.89 | 0.00 | 16.23 | 0.14 | 11.89 | 0.49 |
| Bongo | 0.79 | 0.47 | 34.34 | 0.16 | 3.19 | 2.69 | 2.53 | 0.48 |
| Charly | 8.04 | 5.49 | 17.25 | 2.75 | 2.15 | 0.59 | 14.31 | 0.45 |
| Cheeta | 1.72 | 0.92 | 54.02 | 0.11 | 13.41 | 0.00 | 6.55 | 0.65 |
| Coco | 31.26 | 30.96 | 21.85 | 1.52 | 7.16 | 0.00 | 7.74 | 0.89 |
| Juanito | 2.95 | 0.49 | 33.61 | 0.33 | 8.43 | 0.16 | 10.16 | 0.24 |
| Marco | 3.25 | 1.27 | 28.57 | 0.14 | 5.89 | 0.14 | 9.34 | 0.66 |
| Nico | 3.71 | 3.02 | 54.76 | 0.00 | 6.48 | 0.70 | 2.32 | 0.60 |
| Tico | 0.22 | 0.33 | 65.21 | 1.09 | 4.02 | 0.00 | 0.22 | 0.97 |
| Tom | 1.44 | 0.39 | 58.09 | 0.26 | 9.65 | 0.78 | 6.53 | 0.81 |
| Toni | 0.95 | 0.00 | 36.67 | 0.00 | 2.00 | 0.00 | 6.90 | 0.65 |
| Victor | 0.52 | 5.21 | 55.34 | 6.25 | 9.53 | 0.13 | 2.86 | 0.67 |
| Waty | 15.44 | 14.52 | 21.20 | 0.46 | 7.08 | 0.23 | 26.04 | 0.22 |
| **Mean±SD** | **8.92±15.37** | **7.82±13.96** | **40.78±17.23** | **0.95±1.71** | **7.43±4.08** | **0.41±0.71** | **8.01±6.52** | **0.61±0.22** |
